# Continuum of hepatitis C care in France: A 20-year cohort study

**DOI:** 10.1371/journal.pone.0183232

**Published:** 2017-08-29

**Authors:** Coralie Hermetet, Frederic Dubois, Catherine Gaudy-Graffin, Yannick Bacq, Bernard Royer, Christophe Gaborit, Louis D’Alteroche, Jean Claude Desenclos, Philippe Roingeard, Leslie Grammatico-Guillon

**Affiliations:** 1 SIMEES, CHRU de Tours, Laboratoire de Santé Publique, Université François Rabelais, Tours, France; 2 INSERM U966, Université François Rabelais et CHRU de Tours, Tours, France; 3 Service de Bactériologie-Virologie-Hygiène, CHRU de Tours, Tours, France; 4 UC-IRSA, Département 37, La Riche, France; 5 Service de d'Hépato-gastro-entérologie, CHRU de Tours, Tours, France; 6 Direction Scientifique, InVS, Saint-Maurice, France; 7 Laboratoire de Biologie Cellulaire, CHRU de Tours, Tours, France; Centers for Disease Control and Prevention, UNITED STATES

## Abstract

**Background:**

Hepatitis C virus (HCV)-infected patients require a specific continuum of care (CoC) from HCV screening to treatment. We assessed CoC of HCV-infected patients in a longitudinal study.

**Methods:**

We established a cohort of subjects undergoing HCV screening (high alanine aminotransferase levels or risk factors) during preventive consultations at a French regional medical center from 1993 to 2013. Patients were considered to be HCV-infected if HCV RNA was detected in their serum. CoC was assessed as described by Viner *et al*. (*Hepatology 2015*): Stage 1, HCV screening; Stage 2, HCV RNA testing; Stage 3, continuing care; Stage 4, antiviral treatment. Cox multivariate analysis was performed to identify factors favoring CoC, defined as at least one course of antiviral treatment.

**Results:**

In total, 12,993 HCV tests were performed and 478 outpatients were found to be HCV-seropositive. We included 417 seropositive patients, after excluding false positives and patients lost to follow-up. The baseline characteristics of the patients were: sex ratio (M/F) 1.4; mean age 38.5 years; intravenous drug use (IDU) in 55%; and 28% in unstable social situations, estimated by the EPICES deprivation score. Antiviral treatment was initiated for 179 (42.9%) of the 379 (90.9%) patients attending specialist consultations. CoC was associated with screening after 1997 (HR 2.0, 95%CI 1.4–2.9), age > 45 years (HR 1.5, 95%CI 1.02–2.3), patient acceptance of care (HR 9.3, 95%CI 5.4–16.10), specialist motivation for treatment (HR 10.9, 95%CI 7.4–16.0), and absence of cancer (HR 6.7, 95%CI 1.6–27.9). Other comorbid conditions, such as depression and IDU, were not associated with CoC.

**Conclusions:**

Our 20-year cohort study reveals the real-life continuum of care for HCV-infected patients in France. The number of patients involved in HCV care after positive testing was substantial due to the organization of healthcare in France. An improved CoC along with new direct-acting antivirals should help to decrease chronic HCV infection.

## Introduction

The emergence of hepatitis C virus (HCV) over recent decades has created a major public health burden [1,2], with approximately180 million people around the world infected [3–5]. Acute infection progresses to chronic hepatitis in 55 to 85% of cases, and chronic HCV infection leads to severe complications (end-stage cirrhosis, hepatocellular carcinomas, liver transplants) [3–7]. A 1994 population-based survey estimated the prevalence of HCV infection in France to be 1.1% of the population [8,9]. The French health authorities launched a public-health response including primary prevention, the promotion of HCV screening, access to treatment, and research [10]. In 2004, an apparent decrease in prevalence was reported, with an estimated 250,000 infected people (0.84%)[11]. However, the proportion of HCV-infected individuals who are aware of their infection status is just over 50% [4,7,12], despite the investment in primary prevention, social marketing campaigns to promote screening, and the mobilization of healthcare professionals in France against HCV infection. Silent HCV infection may affect 80,000 people in France that have yet to be tested (former intravenous drug users (IDU) [13,14] and other at-risk groups, such as migrants and prison inmates [15,16]).

The treatment of HCV infection has improved significantly over the last two decades [12], especially with the recent development of direct-acting antiviral drugs (DAA) with shorter treatment duration and fewer side effects than previous drugs. This has considerably improved the prognosis of HCV patients, with cure rates exceeding 90% for all genotypes [17–19]. With these new developments, the eradication of HCV infection is foreseeable [20], perhaps by 2030 [21] if HCV-infected patients can be identified and treated early enough [22,23]. In this context, assessment of the continuum of care (CoC) from infection to cure is of strategic importance for policy implementation and evaluation [7]. For example, several simple, inexpensive operational interventions have been demonstrated to substantially improve engagement along the HCV CoC (e.g., promoting HCV testing and nurse-led educational interventions) [24].

This cohort study, based on a 20-year risk-based screening program within a population health system, provides an overview of the CoC for HCV-infected patients in France, during the different treatment eras, and investigates the factors associated with access to CoC.

## Methods

### Study population

A cohort of HCV-infected individuals was built through a social security screening program implemented at a medical center in France, between 1993 and 2013. This social security medical center, run by the French National Health Insurance System, offers patients with social security coverage, unemployed individuals, and welfare recipients a biomedical examination every five years [8]. The district served by this medical center has 591,000 inhabitants, 85% of whom are covered by the French social security system.

### Subject inclusion

The medical check-up at the medical center includes a series of biological tests, including serum alanine aminotransferase (ALT), followed by a clinical examination. The medical center has established routine testing of serum alanine aminotransferase (ALT) levels as a guide for selective testing for hepatitis C during the medical checkup [8]. Subjects were selected for HCV screening using serological tests for HCV antibodies (Ab) (enzyme immunoassay (EIA) or first- to third-generation assays, depending on the recommendations during the period concerned), as specified in the protocol established and followed by nurses and medical doctors. Every arriving medical fellow, nurse, or practitioner had to be trained before beginning preventive consultation. All individuals with high ALT levels were tested: ALT levels of 1.2-fold, or more, above the normal value according to age and sex [8,25,26]. HCV testing was also performed on patients without high ALT levels, but with risk factors for HCV infection, such as IDU, blood transfusion, and known exposure to HCV. All EIA-positive samples were verified using a third-generation strip immunoblot assay (RIBA HCV strip immunoblot assay, available up to 2007, notably RIBA-3, Chiron Corp., Emeryville, CA, then replaced by inno-LiPa HCV Score, Innogenetics, Ghent, Belgium). All individuals undergoing medical check-ups between 1993 and 2013 and eligible for HCV testing were screened and included in the cohort if the first and control HCV Ab tests were positive.

**Fig 1:**
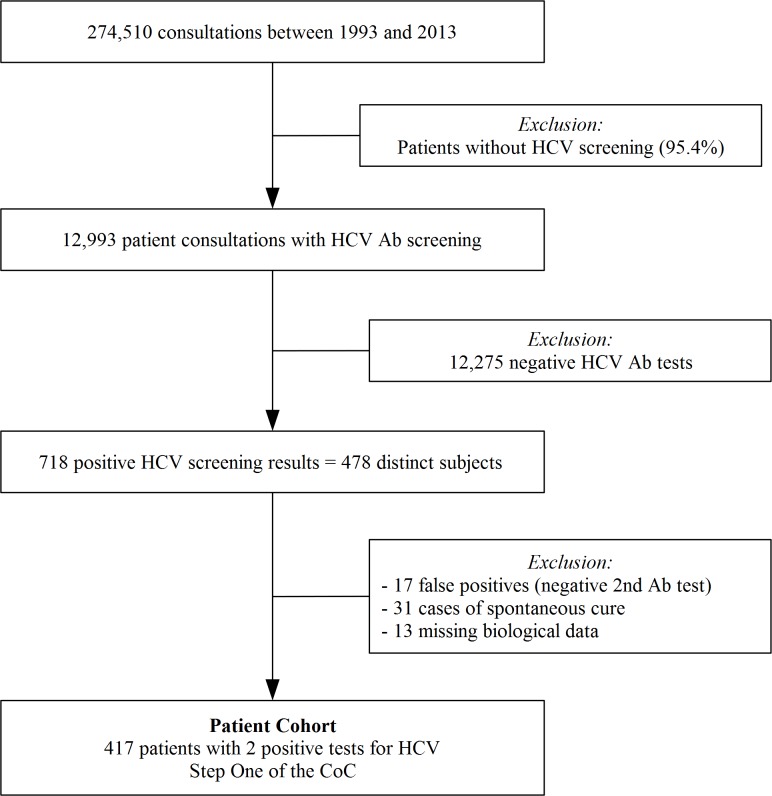
Selection of the study population for assessment of the HCV infection CoC. HCV: Hepatitis C Virus; CoC: continuum of care; Ab: antibody.

During the study period, 274,510 medical check-ups were performed: 12,993 (4.7%) HCV Ab tests were performed with 718 positive cases, corresponding to 478 different patients. We finally included 417 individuals seropositive for HCV by the second Ab test (step 1 of CoC) in the cohort, due to the exclusion of spontaneous HCV cure (*N* = 31) and false-positive HCV Ab test results (Fig 1).

The examination center immediately informed the patient’s GP of a positive HCV Ab test, requiring a specific follow-up by the general practitioner (GP). The confirmatory test was carried out by the patient’s GP, who was responsible for the CoC of the positive HCV patients, especially confirmatory RNA testing and referral to a specialist. Patients were considered to be chronically infected with HCV if a positive result was obtained in an HCV RNA test (test carried out according to the recommendations of the period in a biological laboratory routinely performing the RNA test), corresponding to step 2 of the CoC. Follow-up and outcome variables were obtained for these patients by requesting information from the patient’s GP and/or hepatologist if necessary.

### Data collection

Age, sex, medical history, alcohol, tobacco, and drug abuse, and the deprivation score, based on the French EPICES index [27], were collected at inclusion. Two time periods were defined according to treatment eras: interferon alone up to 1997 (p1); and p2, beginning with the advent of combined treatment with interferon and ribavirin. The HCV genotype was determined when RNA amplification was possible (InnoLipa, HCV Score, Innogenetics, Ghent, Belgium according to current guidelines).

### Outcome and explanatory variables

The HCV CoC was defined as: stage 1, HCV screening; stage 2, positive test for HCV RNA; stage 3, continuing care, defined by a specialist consultation; stage 4, care and antiviral treatment. HCV cure was defined by a prolonged period (> 6 months) of negative PCR results.

The factors associated with CoC were analyzed by constructing two binary variables from the information obtained from medical records or a medical interview: willingness of the physician to treat the patient according to treatment recommendations based on comorbid conditions, medical history, and the patient’s motivation to be cured according to the concerns or reluctance of the patient to start treatment, based on the conclusions drawn by the physician.

### Statistical analysis

The CoC for HCV infection, from stage 1 to 4, was described for all patients included over the 20-year period, then by treatment era. The case fatality rate (overall and directly due to HCV) was calculated as a density rate, with the number of deaths as the numerator and the person-time contribution (time between step 1 and the most recent information for each patient) of all included patients chronically infected with HCV (Step 2) as the denominator.

We performed multivariate analysis of the factors associated with the occurrence of a specialist consultation within one year of screening, using a logistic regression model. The endpoint was the one-year of follow-up, corresponding to a manageable interval between the diagnosis of HCV viremia and counseling.

Kaplan Meier estimates were used to describe access to treatment (Step 4) during follow-up. The endpoint of the survival analyses was December 31, 2013. All possible explanatory variables for consultation occurrence were first tested in a bivariate survival model. Variables of interest, with a *p* value < 0.2 by bivariate analysis were included in the multivariate analysis. Age and sex were systematically included. Cox proportional hazards models were then used to assess the effects of various confounding factors, as well as the time period, on the likelihood of CoC success (Step 3). Hazard ratios (HRs) and their 95% confidence intervals [95%CI] were calculated. We assessed the proportionality of hazards and used the log rank test to compare survival curves.

Statistical analysis was performed using SAS software, version 9.1 (SAS).

### Ethics approval

This study was approved by the CNIL, Commission Nationale de l’Informatique et des Libertés, Paris, France, no. 1739731 and the District CNIL of Tours University Hospital no. 2015_015. All patients included in this study were personally informed by a written document of the treatment of the data, as well as their right to object and access the data, according to articles L.1121-1 and R1121-2 of the French Code of Public Health. The need for individual patient consent was waived by the Research Ethics Committee as the study was considered to be a quality assurance project (authorization no. 2015 013).

## Results

### Characteristics of the study population

We included 417 patients in the cohort (Fig 1), distributed between the treatment periods as follows: 57% up to 1997 and 40% from 1998. The estimated prevalence in the medical center was 3.2%. Data was censured for 19% of patients (N = 78) during the study period due to lost-of-follow-up.

The mean age of the patients at the time of the first test was 38.5 years (range 11–79 years). The sex ratio was 1.38 and 29% of cases were socially deprived. IDU was reported for 55% of the HCV-infected patients. The most frequently identified comorbid conditions were: alcohol abuse, liver diseases, and depression. Only five coinfections with HIV were identified. Less than 5% of the data were missing for almost all variables in the study database, with less than 3% missing for treatment period (N = 12), but 17% for the social deprivation score.

Seventy patients were already aware that they were seropositive for HCV from a previous check-up. HCV genotypes were determined for 172 patients (41%). Genotype 1 (subtypes a and b) was the most common (55%), followed by genotypes 3 (31%) and 4 (8%).

### Characteristics of the continuum of care

The demographic profile of the individuals at each stage of the HCV continuum of care is shown in Table 1. The proportion of male patients was greater among those receiving HCV treatment (stage 4) than among those at earlier stages of the continuum (*p* < 0.05). The proportion of HCV patients did not differ significantly between age groups. Most patients were less than 45 years old, except at stage 2 (more than 55% over 45 years) but this difference was not statistically significant. There were no apparent differences across the continuum for the deprivation index, at-risk group, or first test location. The population of patients at stage 1 was mostly male (83%), as at step 4, whereas the proportion of female patients was higher at stages 2 and 3 (Table 2).

**Table 1:** Baseline characteristics of the patients of the cohort, 1993–2013, Indre-et-Loire, France.

Patient characteristics	Screening period			
	Total *N* (%)	Period 1 *N* (%)	Period 2 *N* (%)	*p*
Total	405 (100)*	238 (57.0)	167 (40.0)	
Sex Female	175 (42)	95 (39.9)	75 (44.9)	NS
Male	242 (58)	143 (60.1)	92 (55.1)	
Age at first positive test (range)	38.5 (11–79)	35.8 (11–73)	42.3 (19–79)	< 0.05
Social deprivation Yes	118 (28.3)	73 (30.6)	43 (25.7)	NS
No	229 (54.9)	127 (53.4)	95 (56.9)	
Missing data	70 (16.8)	38 (16)	29 (17.4)	
Mode of infection IDU	228 (54.7)	132 (55.4)	89 (53.3)	NS
Blood transfusion	87 (20.8)	58 (24.4)	25 (15)	< 0.05
Mixed	6 (1.4)	3 (1.3)	3 (1.8)	NC
Other	82 (19.7)	38 (16)	44 (26.3)	< 0.05
Missing data	14 (3.4)	7 (2.9)	6 (3.6)	NC
First test Examination center	225 (54.0)	99 (41.6)	126 (75.4)	< 0.05
Check-up	70 (16.8)	53 (22.3)	17 (10.2)	< 0.05
Abnormal transaminase results	25 (6.0)	17 (7.1)	7 (4.2)	NS
Medical history	51 (12.2)	38 (16)	11 (6.6)	< 0.05
Clinical symptoms	20 (4.8)	17 (7.1)	3 (1.8)	< 0.05
Missing data	26 (6.2)	14 (5.9)	3 (1.8)	NC
Comorbid conditions Alcohol abuse	86 (20.6)	50 (21)	35 (21)	NS
Depression	60 (14.4)	31 (13)	23 (13.8)	NS
Other liver diseases	49 (11.8)	29 (12.2)	20 (12)	NS
Thyroid diseases	14 (3.4)	9 (3.8)	5 (3)	NS
Hematological diseases	10 (2.4)	5 (2.1)	5 (3)	NS
Cardiovascular diseases	11 (2.6)	8 (3.4)	3 (1.8)	NS
Mental problems	15 (3.6)	9 (3.8)	3 (1.8)	NS
Infectious diseases	10 (2.4)	6 (2.5)	3 (1.8)	NS
Immunological diseases	6 (1.4)	3 (1.3)	3 (1.8)	NS
Neurological diseases	7 (1.7)	3 (1.3)	4 (2.4)	NS
Diabetes mellitus	17 (4.1)	9 (3.8)	8 (4.8)	NS
Non-liver cancers	17 (4.1)	10 (4.2)	7 (4.2)	NS

*Period data missing: N = 12

Period 1: 1993–1997; Period 2: 1998–2013

IDU: intravenous drug use

**Table 2:** Demographics of individuals at of the various stages of the hepatitis C testing to care and treatment.

Demographics		Total *N* = 417 (100%)	Stage of HCV testing and care				
			Stage 1	Stage 2	Stage 3	Stage 4	
			Ab +/-Ag *N* = 12 (%)		In care		*P* value
					Yes		
				Ab+ RNA *N* = 27 (%)	No Antiviral treatment Ab + RNA *N* = 199 (%)	Antiviral treatment Ab + RNA *N* = 179 (%)	
Sex	Male	242 (100)	10 (4.1)	16 (6.6)	101 (41.7)	115 (47.6)	< 0.05
	Female	175 (100)	2 (1.1)	11 (6.3)	98 (56.0)	64 (36.6)	
Age group	≤ 45 years	303 (100)	9 (3.0)	15 (5.0)	142 (46.8)	137 (45.2)	NS
	> 45 years	102 (100)	2 (2.0)	9 (8.8)	50 (49.0)	41 (40.2)	
	Missing data	12 (100)	1 (8.3)	3 (25.0)	7 (58.4)	1 (8.3)	
Deprivation index	Yes	118 (100)	2 (1.7)	6 (5.1)	54 (45.7)	56 (47.5)	NS
	No	229 (100)	8 (3.5)	19 (8.3)	105 (45.8)	97 (42.4)	
	Missing data	70 (100)	2 (2.9)	2 (2.9)	40 (57.1)	26 (37.1)	
At-risk population	IDU	228 (100)	7 (3.1)	16 (7.0)	108 (47.4)	97 (42.5)	NS
	Transfusion	87 (100)	1 (1.2)	3 (3.4)	44 (50.6)	39 (44.8)	
	Mixed	6 (100)	0 (0)	0 (0)	3 (50.0)	3 (50.0)	
	Other	82 (100)	1 (1.2)	7 (8.5)	39 (47.6)	35 (42.7)	
	Missing data	14 (100)	3 (21.4)	1 (7.1)	5 (35.7)	5 (37.7)	
First screening	Medical examination center	225 (100)	8 (3.5)	17 (7.6)	110 (48.9)	90 (40.0)	NS
	Check-up	70 (100)	1 (1.5)	4 (5.7)	30 (42.8)	35 (50.0)	
	Abnormal transaminase results	25 (100)	0 (0)	0 (0)	10 (40.0)	15 (60.0)	
	Medical history	51 (100)	2 (3.9)	2 (3.9)	24 (47.1)	23 (45.1)	
	Clinical symptoms	20 (100)	0 (0)	2 (10.0)	9 (45.0)	9 (45.0)	
	Missing data	26 (100)	1 (3.8)	2 (7.7)	16 (61.6)	7 (26.9)	

Stage 1, HCV screening; Stage 2, positive test for HCV RNA; Stage 3, continuing care, defined by a specialist consultation; Stage 4, care and antiviral treatment; Ab = Antibody

Over the entire study period, most patients reached stage 3 of the CoC (90%), and 43% had at least one antiviral treatment for HCV. During period p1, 64% of the patients reached step 3, with only 10% obtaining treatment within p1. However, 103 (45%) patients included in the first period p1 received treatment for HCV infection during period p2. During p2, 167 patients were included, 90% of whom reached step 3, with 42% obtaining antiviral treatment. The case fatality rate was 7.4/1,000 person-years (31 deaths). Specific case fatality directly due to HCV infection was 4.3/1,000 person-years (18 deaths).

Prolonged remission was observed in 65% of the 179 patients undergoing antiviral treatment during follow-up, mostly (95%) during p2. At the endpoint, 150 patients had been followed, but had not yet received any treatment. However, the doctors responsible for patient care did not consider treatment to be useful for 60% of the patients in regular care, mostly because of the absence of fibrosis/cirrhosis, or the presence of only early stage fibrosis.

### Factors associated with consultation within one year of HCV diagnosis

The factors associated with progression to stage 3 of the CoC within one year of screening by multivariate analysis were: performance of the first positive test at the examination center (OR = 2.2), screening during p2 (OR = 2.6), social deprivation (OR = 3.2), and age over 45 years at the time of screening (OR = 1.9) (Table 3). In contrast, a first positive test for HCV at a check-up and being at-risk were significantly associated with a longer time to specialist consultation.

**Table 3:** Occurrence of a specialist consultation within one year after the first positive test for HCV, assessed by logistic regression (N = 205).

Population characteristics	Individuals		Bivariate analysis	Multivariate analysis
	*N*	%	OR (95% CI)	OR (95% CI)
Sex Female	100	48.7	1	-
Male	105	51.2	0.60 (0.40–0.91)	
Age ≤ 45 years	139	67.8	1	
> 45 years	66	32.2	2.36 (1.44–3.87)	1.88 (1–3.52)
Social deprivation	81	39.5	3.03 (1.84–4.97)	3.16 (1.83–5.44)
At-risk population				
IDU	102	49.8	0.51 (0.33–0.78)	-
Blood transfusion	55	26.8	1.67 (1.01–2.76)	-
Other	51	24.9		
First positive screening				
At the medical examination center	141	68.8	2.95 (1.92–4.54)	2.18 (1.32–3.61)
Check-up	23	11.2	0.36 (0.21–0.63)	0.36 (0.19–0.68)
Increase in ALT levels	10	4.9		
Risk factors	17	8.3	0.44 (0.23–0.83)	0.40 (0.19–0.86)
Clinical symptoms	10	4.9		
Screening period				
1993 to 1997	95	43.3	1	
1998 to 2013	110	53.7	2.77 (1.81–4.24)	2.55 (1.50–4.33)
Comorbid conditions				
Alcohol dependence	36	17.6	0.57 (0.36–0.96)	-
Depression	26	12.7		
Other liver diseases	23	11.2		
Non-liver cancers	11	5.4		
Psychiatric disorders	4	1.9		

### Factors associated with the initiation of antiviral treatment

**Fig 2:**
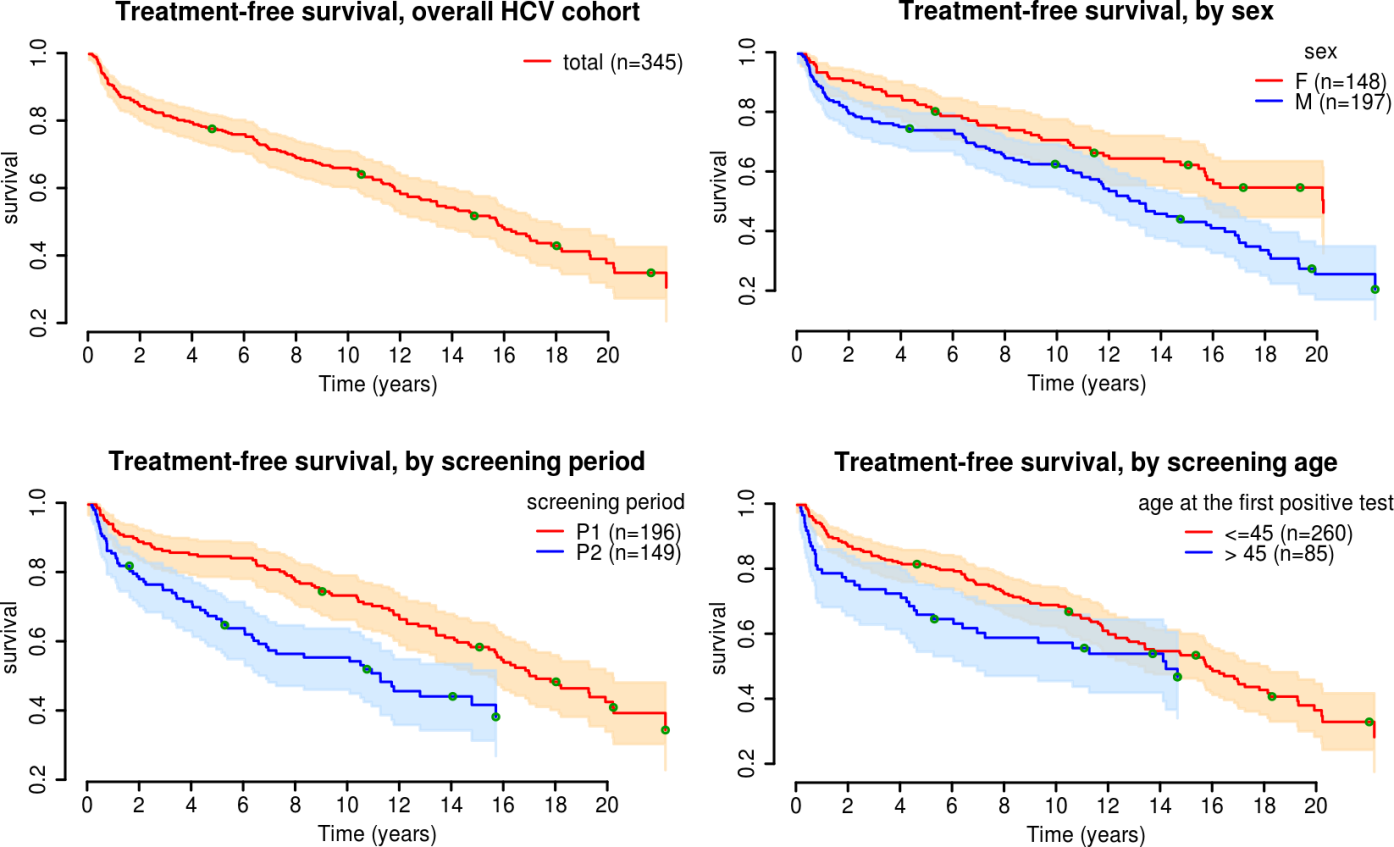
Kaplan-Meier curves for CoC up to step 4 (antiviral treatment), by sex, period, and age: 1993–2013. HCV: Hepatitis C Virus;CoC: continuum of care of HCV-infected patients between HCV testing and antiviral treatment; M: male; F: female P1: screening period 1993–1997; P2: screening period 1998–2013.

We plotted Kaplan-Meier curves for the CoC (Fig 2). Approximately 15% of patients were treated in the first two years after inclusion (censored data corresponded to 50% of the total cohort). Male patients, patients screened during period 2, and patients over the age of 45 years had shorter intervals between screening and the occurrence of stage 4 of the CoC (Fig 2). Approximately 70% of the patients reaching stage 3 had a consultation with a specialist during the first two years after HCV screening. Female patients, patients screened during period 2, and older patients reached stage 3 more rapidly. We found that 20% of patients obtained treatment within two years of specialist consultation. Men were treated earlier than women and patients screened during p2 were treated more rapidly than those screened during period 1, whereas age at the first HCV positive test did not seem to be linked to the absence of CoC progression (Fig 2).

In the Cox model, the identification of factors associated with treatment occurrence in the bivariate analysis was performed on 345 patients, whereas the multivariate analysis was based on the 319 patients without missing data for the explicative variables (Table 4). Hence, the factors significantly associated with a first antiviral treatment were age over 45 years (HR 1.54), screening after 1997, absence of cancer, the physician’s view that treatment was necessary (HR 10.0), and the patient’s willingness to be treated (HR 9.3) (Table 4).

**Table 4:** Multivariate analysis (Cox model) of antiviral treatment initiation after HCV diagnosis.

Population characteristics	Individuals		Bivariate analysis	Multivariate analysis (N = 319)
	*N = 345*	(100%)	HR (95% CI)	HR (95% CI)
Sex Female	148	42.9	1 2.03 (1.36–3.02)	-
Male	197	57.1		-
Age ≤ 45 years	260	75.4	1 1.20(0.78–1.85)	1
> 45 years	85	24.6		1.54 (1.02–2.31)
Social deprivation *missing data*	106 *57*	36.8	1.25 (0.85–1.87)	-
At-risk population				
IDU			0.84 (0.58–1.22)	-
Blood transfusion	78	22.6	0.93 (0.61–1.42)	-
Other	71	20.6	1.43 (0.93–2.21)	-
First positive screening				
At medical examination center	194	56.2	2.35 (1.52–3.34)	
Check-up	61	17.7	0.57 (0.32–1.01)	
Increase in ALT levels	21	6.1	1.09 (0.53–2.24)	
Risk factor			1.60(0.61–4.17)	
Clinical symptoms	16	4.6	0.39 (0.09–1.56)	
Screening period				
1993 to 1997	196	56.8	1 1.82 (1.31–2.52)	1
1998 to 2013	149	43.2		1.97 (1.36–2.85)
Comorbidity				
Alcohol dependence	76	22.0	1.06 (0.69–1.69)	-
Depression	49	14.2	0.81 (0.47–139)	-
Other liver diseases	47	13.6	0.75 (0.41–1.37)	-
Absence of cancer	329	95.4	2.62 (0.83–8.26)	6.67 (1.60–27.9)
No psychiatric disorders	336	97.4	4.21 (0.59–30.23)	-
Willingness of physician to initiate antiviral treatment	210	60.9	3.86 (2.77–5.37)	10.86 (7.38–15.97)
Willingness of the patient to be treated	81	23.5	2.99 (1.84–4.85)	9.30 (5.42–15.97)

## Discussion

This is the first 20-year study of the CoC for chronic HCV infection to be carried out at the population level in France. We found that 3.2% of subjects targeted for HCV screening from the French general population had HCV infection. This is three-fold higher than other French prevalence estimations based on targeted populations. Over two decades of the study, the frequency of anti-HCV antibodies in this population decreased, HCV screening activity increased substantially, and the proportion of HCV tests yielding positive results decreased markedly after 2000. A substantial number of patients who tested positive for HCV entered the CoC (stages 3 and 4 accounted for 90% of HCV-infected patients), showing the positive impact of this targeted operational intervention, with a higher proportion of patients receiving counseling and treatment in the second decade. Thus, this public health screening intervention may have expedited progression through the CoC, improving engagement along the HCV CoC (e.g., by alerting GPs of a positive test), as recently demonstrated in a systematic review and meta-analysis for viral hepatitis CoC [24].

Understanding the reasons for patients entering the CoC is critical for modifying public health policy and physician practices to increase the number of patients treated. Our findings highlighted key points for progression through the CoC. Effective organization facilitates progression through a CoC and health policies that favor HCV screening of the at-risk population improve the CoC (e.g., the timely identification of at-risk patients due to targeted screening) which must be further enhanced in the near future with the availability of new active antivirals.

Advances in HCV therapy have ushered in a new era in chronic hepatitis treatment [24]. Individuals must be engaged and retained in care to maximize the effects of the new DAAs [5,7,12,20,28,29]. Our French medical center appears to effectively promote progression through the CoC, with 43% of patients reaching one antiviral treatment by the end of our study versus only 27% in a recent US study [7]. The varying results between the French and US studies may be accounted for by differences in HCV screening policies between the two countries. First, our protocol associated the medical questionnaire with the medical interview performed in a two-step sequence (the first by a nurse and the second by a medical doctor, both trained in the HCV screening protocol), associated with the biological data (ALT levels) detected most concerned individuals. Second, this could be due to the effective organization at the examination center, which carries out centralized HCV testing and immediately informs the patient’s GP to facilitate testing for the presence of HCV RNA. Third, the long study period, with patients coming back several times to the center, helped practitioners convince the patients to consult their GP and begin the CoC with a specialist. These findings suggest that targeted screening, based on a questionnaire addressing only the most common risk factors, such as that used by the social security medical examination center, may be of little added value if the physicians involved in the testing procedure are not sufficiently well trained, as recently shown in the international literature [8,26]. Our testing procedure corresponds to the approach currently proposed by the American authors to US health policy-makers [7]. Too few at-risk patients are appropriately screened in the USA and too few infections are confirmed and managed, despite the recommendations for HCV screening.

A large part of the better progression through the CoC in France may also be explained by the French public health policy, especially the reimbursement of medical care costs by the French National Health Insurance. Finally, these key elements of the CoC, along with the current DAA era, aid the promotion of HCV screening in at-risk populations and better professional coordination of healthcare.

This study has some limitations. Our population was composed of apparently at-risk people targeted for HCV screening, giving higher HCV prevalence. The General population was not represented. However, the association of the medical questionnaire and biological data in our protocol helped to detect most of the concerned individuals, even if any investigation based on a risk factor screening method may miss some patients. Moreover, our cohort demonstrated a protocol for testing and following at-risk subjects for hepatitis C. Furthermore, this study lacked some information, due to missing data, such as the stage of fibrosis. However, we analyzed a very large population, providing reliable data on the French at-risk population. Indeed, our report is the largest study of the CoC of Hepatitis C in a French population.

In conclusion, our population-based cohort study reveals the real-life CoC for HCV-infected patients in a French district. The number of patients involved in HCV care after positive testing was substantial, due to focused screening and a good organization of healthcare. An improved CoC, along with new direct-acting antivirals, should help to decrease chronic HCV infection and its complications, thereby increasing survival and reducing the burden of HCV infection.

## Supporting information

S1 Fig Distribution of care continuum stages for HCV-positive patients in *Indre-et-Loire*, 1993–2013 (N = 417). S1a: Continuum of care during p1 (1993–1997) for patients screened during p1S1b: Continuum of care during p2 (1998–2013) for patients screened during p1S1c: Continuum of care during p2 (1998–2013) for patients screened during p2S1d: Continuum of care during p2 for patients screened during p1 or p2 (1993–2013).
(JPG)

S2 Fig Kaplan-Meier curves for CoC up to step 3 (specialist consultation), by sex, period, and age: 1993–2013. M: male; F: female.
(TIFF)

S1 Dataset HCV cohort database. (XLSX)

## References

[1] Shepard CW, Finelli L, Alter MJ. Global epidemiology of hepatitis C virus infection. Lancet Infect Dis. 2005;5: 558–567. doi: 10.1016/S1473-3099(05)70216-4 16122679 10.1016/S1473-3099(05)70216-4

[2] Marcellin P, Asselah T, Boyer N. Fibrosis and disease progression in hepatitis C. Hepatology. 2002;36: S47–S56. doi: 10.1053/jhep.2002.36993 12407576 10.1053/jhep.2002.36993

[3] Chen SL, Morgan TR. The natural history of hepatitis C virus (HCV) infection. Int J Med Sci. 2006;3: 47–52. 16614742 10.7150/ijms.3.47 PMC1415841

[4] Delarocque-Astagneau E, Meffre C, Dubois F, Pioche C, Le Strat Y, Roudot-Thoraval F, et al The impact of the prevention programme of hepatitis C over more than a decade: the French experience. J Viral Hepat. 2010;17: 435–443. doi: 10.1111/j.1365-2893.2009.01196.x 19780936 10.1111/j.1365-2893.2009.01196.x

[5] Dhumeaux D, Marcellin P, Lerebours E. Treatment of hepatitis C. The 2002 French consensus. Gut. 2003;52: 1784–1787. 14633963 10.1136/gut.52.12.1784 PMC1773901

[6] Smith BD, Morgan RL, Beckett GA, Falck-Ytter Y, Holtzman D, Teo C-G, et al Recommendations for the identification of chronic hepatitis C virus infection among persons born during 1945–1965. MMWR Recomm Rep. 2012;61: 1–32. 22895429

[7] Viner K, Kuncio D, Newbern EC, Johnson CC. The continuum of hepatitis C testing and care. Hepatology. 2015;61: 783–789. doi: 10.1002/hep.27584 25348499 10.1002/hep.27584

[8] Dubois F, Desenclos JC, Mariotte N, Goudeau A. Hepatitis C in a French population-based survey, 1994: seroprevalence, frequency of viremia, genotype distribution, and risk factors. The Collaborative Study Group. Hepatology. 1997;25: 1490–1496. doi: 10.1002/hep.510250630 9185773 10.1002/hep.510250630

[9] Meffre C, Le Strat Y, Delarocque-Astagneau E, Dubois F, Antona D, Lemasson J-M, et al Prevalence of hepatitis B and hepatitis C virus infections in France in 2004: social factors are important predictors after adjusting for known risk factors. J Med Virol. 2010;82: 546–555. doi: 10.1002/jmv.21734 20166185 10.1002/jmv.21734

[10] Brody H. Hepatitis C. Nature. 2011;474: S1–S1. doi: 10.1038/474S1a 21666726 10.1038/474S1a

[11] Mohd Hanafiah K, Groeger J, Flaxman AD, Wiersma ST. Global epidemiology of hepatitis C virus infection: new estimates of age-specific antibody to HCV seroprevalence. Hepatology. 2013;57: 1333–1342. doi: 10.1002/hep.26141 23172780 10.1002/hep.26141

[12] Pol S, Corouge M. Treatment of hepatitis C: Perspectives. Med Mal Infect. 2014; doi: 10.1016/j.medmal.2014.07.015 25174659 10.1016/j.medmal.2014.07.015

[13] Edlin BR. Hepatitis C screening: getting it right. Hepatology. 2013;57: 1644–1650. doi: 10.1002/hep.26194 23239521 10.1002/hep.26194 PMC4751877

[14] Marcellin P, Pequignot F, Delarocque-Astagneau E, Zarski J-P, Ganne N, Hillon P, et al Mortality related to chronic hepatitis B and chronic hepatitis C in France: evidence for the role of HIV coinfection and alcohol consumption. J Hepatol. 2008;48: 200–207. doi: 10.1016/j.jhep.2007.09.010 18086507 10.1016/j.jhep.2007.09.010

[15] Almasio PL, Babudieri S, Barbarini G, Brunetto M, Conte D, Dentico P, et al Recommendations for the prevention, diagnosis, and treatment of chronic hepatitis B and C in special population groups (migrants, intravenous drug users and prison inmates). Dig Liver Dis. 2011;43: 589–595. doi: 10.1016/j.dld.2010.12.004 21256097 10.1016/j.dld.2010.12.004

[16] Negro F. Epidemiology of hepatitis C in Europe. Dig Liver Dis. 2014;46 Suppl 5: S158–164. doi: 10.1016/j.dld.2014.09.023 25453870 10.1016/j.dld.2014.09.023

[17] Lawitz E, Poordad FF, Pang PS, Hyland RH, Ding X, Mo H, et al Sofosbuvir and ledipasvir fixed-dose combination with and without ribavirin in treatment-naive and previously treated patients with genotype 1 hepatitis C virus infection (LONESTAR): an open-label, randomised, phase 2 trial. Lancet. 2014;383: 515–523. doi: 10.1016/S0140-6736(13)62121-2 24209977 10.1016/S0140-6736(13)62121-2

[18] Mishra P, Murray J, Birnkrant D. Direct-acting antiviral drug approvals for treatment of chronic hepatitis C virus infection: Scientific and regulatory approaches to clinical trial designs. Hepatology. 2015;62: 1298–1303. doi: 10.1002/hep.27880 25953139 10.1002/hep.27880

[19] Noell BC, Besur SV, deLemos AS. Changing the face of hepatitis C management—the design and development of sofosbuvir. Drug Des Devel Ther. 2015;9: 2367–2374. doi: 10.2147/DDDT.S65255 25987834 10.2147/DDDT.S65255 PMC4422286

[20] Bansal S, Singal AK, McGuire BM, Anand BS. Impact of all oral anti-hepatitis C virus therapy: A meta-analysis. World J Hepatol. 2015;7: 806–813. doi: 10.4254/wjh.v7.i5.806 25914781 10.4254/wjh.v7.i5.806 PMC4404386

[21] Rapport_Prise_en_charge_Hepatites_2014-DHUMEAUX.pdf [Internet]. Available: http://www.sante.gouv.fr/IMG/pdf/Rapport_Prise_en_charge_Hepatites_2014.pdf

[22] Brouard C, Le Strat Y, Larsen C, Jauffret-Roustide M, Lot F, Pillonel J. The undiagnosed chronically-infected HCV population in France. Implications for expanded testing recommendations in 2014. PLoS ONE. 2015;10: e0126920 doi: 10.1371/journal.pone.0126920 25961575 10.1371/journal.pone.0126920 PMC4427442

[23] Vermeiren APA, Dukers-Muijrers NHTM, van Loo IHM, Stals F, van Dam DW, Ambergen T, et al Identification of hidden key hepatitis C populations: an evaluation of screening practices using mixed epidemiological methods. PLoS ONE. 2012;7: e51194 doi: 10.1371/journal.pone.0051194 23236452 10.1371/journal.pone.0051194 PMC3517446

[24] Zhou K, Fitzpatrick T, Walsh N, Kim JY, Chou R, Lackey M, et al Interventions to optimise the care continuum for chronic viral hepatitis: a systematic review and meta-analyses. The Lancet Infectious Diseases. doi: 10.1016/S1473-3099(16)30208-0 10.1016/S1473-3099(16)30208-0 27615026

[25] Desenclos JC, Dubois F, Mariotte N, Goudeau A. [Should hepatitis C be screened? Analysis of oriented screening strategies for hepatitis C virus infection]. Gastroenterol Clin Biol. 1997;21: S25–32. 9161511

[26] Gaudy C, Thevenas C, Tichet J, Mariotte N, Goudeau A, Dubois F. Usefulness of the hepatitis C virus core antigen assay for screening of a population undergoing routine medical checkup. J Clin Microbiol. 2005;43: 1722–1726. doi: 10.1128/JCM.43.4.1722-1726.2005 15814991 10.1128/JCM.43.4.1722-1726.2005 PMC1081371

[27] Sass et al. InVS | BEH n°14 (4 avril 2006). Le score Epices: un score individuel de précarité. Construction du score et mesure des relations avec des données de santé, dans une population de 197 389 personnes. [Internet]. [cited 25 Aug 2015]. Available: http://www.invs.sante.fr/beh/2006/14/

[28] Chavalitdhamrong D, Tanwandee T. Long-term outcomes of chronic hepatitis C patients with sustained virological response at 6 months after the end of treatment. World J Gastroenterol. 2006;12: 5532–5535. doi: 10.3748/wjg.v12.i34.5532 17006994 10.3748/wjg.v12.i34.5532 PMC4088239

[29] Afdhal N, Reddy KR, Nelson DR, Lawitz E, Gordon SC, Schiff E, et al Ledipasvir and sofosbuvir for previously treated HCV genotype 1 infection. N Engl J Med. 2014;370: 1483–1493. doi: 10.1056/NEJMoa1316366 24725238 10.1056/NEJMoa1316366

