# Supplementary figures and images for: Continuum of hepatitis C care in France: A 20-year cohort study
Source: PLoS One. 2017 Aug 29;12(8):e0183232. doi: 10.1371/journal.pone.0183232 (PMC5574535; doi:10.1371/journal.pone.0183232)

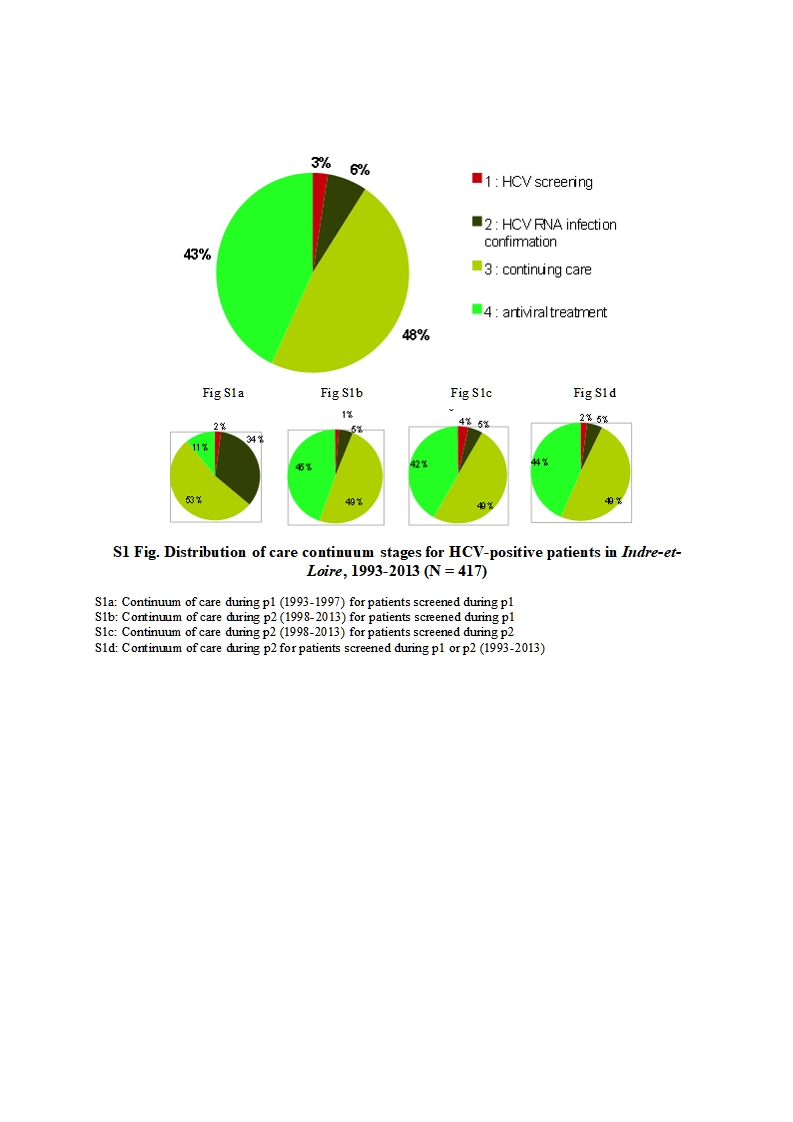

Supplement: S1 Fig — S1a: Continuum of care during p1 (1993–1997) for patients screened during p1S1b: Continuum of care during p2 (1998–2013) for patients screened during p1S1c: Continuum of care during p2 (1998–2013) for patients screened during p2S1d: Continuum of care during p2 for patients screened during p1 or p2 (1993–2013). (JPG) [file pone.0183232.s001.jpg]

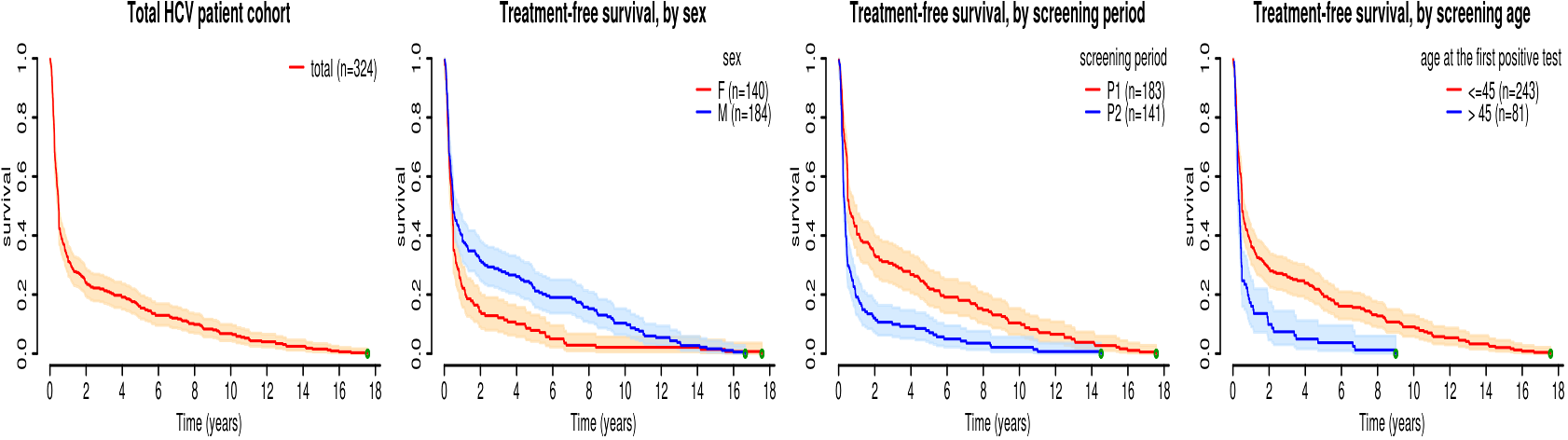

Supplement: S2 Fig — M: male; F: female. (TIFF) [file pone.0183232.s002.tiff]
